# Rationale and limitations of the SpO_2_/FiO_2_ as a possible substitute for PaO_2_/FiO_2_ in different preclinical and clinical scenarios

**DOI:** 10.5935/0103-507X.20220013-en

**Published:** 2022

**Authors:** Eduardo Butturini de Carvalho, Thiago Ravache Sobreira Leite, Raquel Ferreira de Magalhães Sacramento, Paulo Roberto Loureiro do Nascimento, Cynthia dos Santos Samary, Patrícia Rieken Macedo Rocco, Pedro Leme Silva

**Affiliations:** 1 Laboratory of Pulmonary Investigation, Institute of Biophysics Carlos Chagas Filho, Universidade Federal do Rio de Janeiro - Rio de Janeiro (RJ), Brazil.; 2 Veterinary Medicine School, Universidade Federal Fluminense - Niterói (RJ), Brazil.

**Keywords:** COVID-19, SARS-CoV-2, Oxygen saturation, Oximetry, Blood gas analysis, Oxygen, Respiratory distress syndrome, Respiratory insufficiency, Prognosis, Infant, Infant newborn, Adult

## Abstract

Although the PaO_2_/FiO_2_ derived from arterial blood gas analysis remains the gold standard for the diagnosis of acute respiratory failure, the SpO2/FiO2 has been investigated as a potential substitute. The current narrative review presents the state of the preclinical and clinical literature on the SpO_2_/FiO_2_ as a possible substitute for PaO_2_/FiO_2_ and for use as a diagnostic and prognostic marker; provides an overview of pulse oximetry and its limitations, and assesses the utility of SpO_2_/FiO_2_ as a surrogate for PaO_2_/FiO_2_ in COVID-19 patients. Overall, 49 studies comparing SpO_2_/FiO_2_ and PaO_2_/FiO_2_ were found according to a minimal search strategy. Most were conducted on neonates, some were conducted on adults with acute respiratory distress syndrome, and a few were conducted in other clinical scenarios (including a very few on COVID-19 patients). There is some evidence that the SpO_2_/FiO_2_ criteria can be a surrogate for PaO2/FiO2 in different clinical scenarios. This is reinforced by the fact that unnecessary invasive procedures should be avoided in patients with acute respiratory failure. It is undeniable that pulse oximeters are becoming increasingly widespread and can provide costless monitoring. Hence, replacing PaO_2_/FiO_2_ with SpO_2_/FiO_2_may allow resourcelimited facilities to objectively diagnose acute respiratory failure.

## INTRODUCTION

Acute respiratory failure (ARF) is a ubiquitous issue in emergency departments (EDs), operating rooms (ORs) and intensive care units (ICUs) worldwide. Nevertheless, in many health care settings, such as prolonged field care and aeromedical evacuation,^(1)^ arterial blood gas (ABG) analysis—which is required for an objective ARF diagnosis—is unavailable.^(2,3)^ The COVID-19 pandemic has highlighted the urgency of developing rapid, affordable, and easily accessible ARF diagnostics, during the period when timely and appropriate management can have an impact on morbidity and mortality.

Although the ratio of the partial pressure of arterial oxygen to the fraction of inspired oxygen (PaO2/ FiO2), or P/F, derived from ABG analysis remains the gold standard for ARF diagnosis, the arterial blood oxygen saturation to the FiO2 ratio (SpO2/FiO2), or S/F, has been investigated as a potential surrogate.^(4-8)^ Replacement of PaO2 with SpO2 has shown promising results in other areas, such as the oxygenation index, used to assess the severity of hypoxic respiratory failure (HRF) in neonates.^(9)^

Since the first investigations correlating S/F and P/F, few studies have been published in this field. Some of these studies have used S/F as a substitute for the P/F in patients with acute respiratory distress syndrome (ARDS).^(4)^ Furthermore, S/F has been successfully used to impute P/F during Sequential Organ Failure Assessment (SOFA) score evaluation and in other scoring systems,^(10,11)^ and it has even been investigated in trauma and COVID-19.^(3,12)^ This narrative review will discuss the current state of the literature on the S/F, focusing on preclinical and clinical studies investigating it as a possible substitute for the P/F. In addition, an overview of pulse oximetry and its limitations will be provided. Finally, the potential utility of the S/F as a surrogate for the P/F in the particular circumstances of the COVID-19 pandemic will be assessed.

## METHODS

### Literature search strategy

The PubMed*®*, Cochrane Library, and SciELO databases were searched for preclinical and clinical studies evaluating the S/F and its association with the P/F, with no date or language restrictions. The following search queries were used, all with Boolean operators: oximetry AND S/F; oximetry AND P/F; oximetry AND SpO2/ FiO2; oximetry AND PaO2/FiO2; oximetry AND FiO2; S/F AND P/F; SpO2/FiO2 AND PaO2/FiO2. Studies were eligible if they investigated the following aspects: pulse oximeter functioning and artifacts; pulse oximetry values under different inspired oxygen fractions; and any aspect related to S/F correlation. PICO questions were investigated as follows: Patient - sample size and patient characteristics (age and disease); Intervention - if receiving invasive or noninvasive mechanical ventilation (MV) or under spontaneous breathing; Comparison - correlation or regression between S/F and P/F and Outcome - association with survival, ventilator or intensive care-free days and length of stay.

## AN OVERVIEW OF PULSE OXIMETRY

### Rationale

Arterial blood oxygen saturation is one of the oldest monitoring measures in ICUs, EDs, and ORs. Since the ear oximeter was developed by Millikan in 1947 and improved by Aoyagi in 1970, pulse oximetry has gained importance in patient monitoring and is now a widespread technology.^(2,13-15)^ Considering its clinical utility, every health care provider should have at least a basic understanding of pulse oximetry.^(14)^

Arterial blood oxygen saturation monitors calculate blood saturation levels, *i.e.*, the ratio of oxygen-bound hemoglobin (Hb) to unbound Hb in the arterial blood compartment.^(16,17)^ Using an LED light that emits fixed and selected wavelengths, pulse oximeters are equipped with a photodiode that quantifies light transmitted through a tissue based on Beer-Lambert’s law of light absorption, *i.e.*, A = ɛ × b × c, where A is absorbance, ɛ is the absorption (or extinction) coefficient of Hb at a specific wavelength, b is the length of the path that the emitted light travels through the vessel, and c is the Hb concentration.^(14,16)^ Pulse oximeters were previously calibrated using ABG samples from healthy subjects analyzed by a hemoximeter.^(18)^ Pulse oximetry is rarely contraindicated, although it has some limitations that must be understood to avoid pitfalls in interpreting SpO2 values, such as skin color. Two cohorts showed an approximate frequency three times higher that of occult hypoxemia (an arterial oxygen saturation < 88% despite oxygen saturation of 92 to 96% on pulse oximetry) in black patients when compared to white patients, suggesting that other variables should be used for the diagnosis of hypoxemia and the titration of supplementary oxygen levels.^(19)^ Table 1 summarizes the main limitations.

**Table 1:** Main limitations of pulse oximetry

Limitation	Characteristics	References
Anemia	Anemia may cause underestimation of SaO2 by SpO2 readings in hypoxemic patients. SpO2 can accurately represent SaO2 values in hematocrits as low as 10 - 14% in dogs	Zeserson et al.,^(8)^ Hafen et al.,^(14)^ Schnapp et al.,^(15)^ Chan et al.,^(16)^ Lee et al.,^(20)^ Jay et al.,^(21)^ Perkins et al.^(22)^ and Severinghaus et al.^(23)^
Carbon monoxide intoxication	In the presence of carboxyhemoglobin, pulse oximeters consistently overestimate SpO2	Schnapp et al.^(15)^ and Barker et al.^(24)^
Methemoglobinemia	SpO2 readings may decrease when methemoglobin levels rise, but when the latter reach 30 - 35%, PO readings reach a plateau of 80 - 85%	Schnapp et al.,^(15)^ Barker et al.^(25)^ and Eisenkraft^(26)^
High venous pressure	High venous pressure, for example in right heart systolic insufficiency or tricuspid valve regurgitation, may cause falsely low SpO2 values	Zeserson et al.,^(8)^ Bucher et al.,^(27)^ Fouzas et al.^(28)^ and Stewart et al.(29)
Dyes and pigments	Indigo carmine, indocyanine green, and methylene blue may alter SpO2 readings, since they cause tissue pigmentation, thereby altering light absorbance	Schnapp et al.^(15)^ and Fouzas et al.^(28)^
Excessive motion	Artifacts caused by excessive motion can be interpreted as pulse signals and increase the noise-to-signal ratio	Schnapp et al.^(15)^ and Louie et al.^(30)^
Hyperbilirubinemia	Although a case series reported overestimation of SaO2 by pulse oximeters in cirrhotic patients with hyperbilirubinemia, bilirubin has a different light absorption spectrum	Fouzas et al.,^(28)^ Nilles et al.,^(31)^ Salyer^(32)^ and Veyckemans et al.^(33)^
Hyperoxemia/hyperoxia	Pulse oximeters cannot detect hyperoxemia/hyperoxia, yet these events may evoke unwanted responses such as a decrease in cardiac output and heart rate (approximately 10%), 20% reduction in regional blood flow (cerebral, cardiac, skin, and skeletal muscle vascular beds), and a buildup of reactive oxygen species in the mitochondria, causing oxidative stress	Allardet-Servent et al.^(34)^ and Sjöberg et al.^(35)^
Low perfusion	Low perfusion due to hypovolemia, hypothermia, use of vasopressors, and peripheral vascular disease may lead to poor sensor readings, increasing the noise-to-signal ratio	Schnapp et al.^(15)^ and Fouzas et al.^(28)^
External light sources	Although new pulse oximetry equipment can detect excessive light artifacts, there are reports of external light sources flooding the photodetector. Covering the sensor with an opaque material may prevent misreading	Schnapp et al.,^(15)^ Fouzas et al.^(28)^ and Manheimer^(36)^
Skin color	Occult hypoxemia (an arterial oxygen saturation of < 88% despite an oxygen saturation of 92 to 96% on pulse oximetry) was more common in Black (11.7%; 95%CI, 8.5 to 16.0) compared to White patients (3.6%; 95%CI, 2.7 to 4.7)	Sjöberg et al.^(35)^

SaO2 - arterial hemoglobin oxygen saturation; SpO2 - oxygen saturation; PO - pulse oximetry; 95%CI - 95% confidence interval.

Although the precise normal range of SpO2 values is still a matter of debate, it is widely proposed that baseline SpO2 values for spontaneously breathing patients on room air should be interpreted as follows: > 97%, normal lung function; 91 - 96%, slightly to moderately abnormal lung function; and < 90%, hypoxemia (indicating a shunt effect). During MV with FiO2 = 1, normal SpO2 should always be 100%.^(37)^

### Pulse oximetry as an everyday affordable monitoring technology

In the last few years, pulse oximeters have become available not only in health care settings but also to the general public as wearable gadgets. Fingertip oximeters can be purchased in pharmacies and retail stores without a prescription, although their availability has become limited since the COVID-19 pandemic.^(17)^ This shortage may indicate that pulse oximeters could be taking on a growing role in nonhealthcare settings, as blood pressure monitors did before them.

Oximeters embedded in mobile phones and smartwatches have shown variable levels of accuracy across devices. Three iPhone apps that allegedly could give precise SpO_2_ values were proven unreliable in a recent study.^(38)^ This is also an important issue with portable, low-cost fingertip pulse oximeters, some of which demonstrate highly inaccurate readings.^(39)^ Nevertheless, many studies have shown a good correlation between standard oximeters and smartphone-based oximeters.^(17,40-43)^ When the user’s SpO_2_ is > 90%, these devices generally provide good accuracy, creating the possibility for early detection of silent hypoxemia and reduction of ICU admissions, intubations, and mortality.^(17)^

Pulse oximetry is unquestionably gaining ground in nonhealthcare settings and becoming an affordable monitoring technology. It is probably only a matter of time until the accuracy issues are addressed, and S/F may eventually be available on smartphones and smartwatches.

### Why pulse oximeters?

It is always better to avoid invasive procedures when possible. As economists say, “There is no free lunch”. Arterial blood gas carries risks and contraindications of its own; it is a painful procedure and demands technical skills.^(8,44)^ Pulse oximetry has been recognized as a useful tool to detect hypoxemia in underresourced facilities lacking blood gas analysers.^(2)^ Even when blood gas analyzers are available, venous blood gas (VBG) analysis combined with SpO2 could be an easier option. Together, SpO2 and VBG analysis could provide useful information about acid-base, ventilation, and oxygenation status in ICU patients.^(8)^ The S/F allows for continuous “on-screen” respiratory function monitoring. Last but not least, as a relatively old technology, pulse oximeters are much cheaper and more readily available than blood gas analyzers.

### CAN WE RELY ON THE CORRELATION BETWEEN S/F AND P/F?

One of the most important issues is whether there is an acceptable correlation between the S/F and P/F. To date, a small yet promising body of evidence has been published. First, it is imperative to discuss preclinical evidence for the role of SpO2 in predicting or even replacing PaO2 in different scenarios. One study conducted in dogs tried to predict PaO2 from SpO2 using the oxygen-Hb dissociation curve. However, it showed only a slight correlation (0.49 in room air breathing dogs and 0.74 in ventilated dogs, both p < 0.0001).^(45)^ Below a PaO2 of 60mmHg, small reductions in blood oxygen are followed by extreme SpO2 reductions, explained by the sigmoid portion of the oxygen-Hb dissociation curve. In summary, three studies in canine models found P/F-to-S/F correlations ranging from 0.76 to 0.95.^(5,45,46)^

The P/F presents a nonlinear relationship with FiO_2_ at lower shunt levels.^(47)^ In this line, with a 20% shunt, the P/F varies considerably with changes in FiO2. At inferior and superior bounds of FiO2, the P/F is substantially greater than at intermediate FiO2. In addition, prolonged exposure to high FiO2 levels may influence the intrapulmonary shunt fraction due to absorption atelectasis. In acute hypoxemic respiratory failure patients, the P/F is more stable at FiO2 ≥ 0.5 and PaO2 ≤ 100mmHg, common values observed in clinical conditions.^(47)^ Although still not thoroughly investigated, this behavior should also be expected when analyzing the correlation between the latter and the S/F.

## CLINICAL STUDIES: CURRENT STATE AND FUTURE APPLICATIONS

### Neonatal and pediatric clinical studies

Table 2 summarizes the clinical studies according to the PICO criteria. Since ABG is a harsh procedure for neonates and children, S/F have been investigated as a surrogate for P/F in this subset of patients. Certainly, when an arterial blood line is required to monitor the mean arterial pressure or measure the partial pressure of carbon dioxide (PaCO2), a discussion regarding the replacement of P/F by S/F makes no sense. However, seeking to reduce the need for indwelling arterial catheters to measure the oxygenation index (mean airway pressure × FiO2 × 100/PaO2) and objectively diagnose HRF and persistent pulmonary hypertension in neonates, Rawat and colleagues successfully replaced PaO2 with SpO2, noting a correlation coefficient of 0.95.^(9)^ In another study of children with ARDS, SpO2-derived markers were found to be adequate surrogates for those using PaO2 when SpO2 is between 80 and 97%.^(48)^ Using the standard oxygen-Hb dissociation curve, a cohort study of children with ARF showed that, approximately 95% of the time, an SpO2 of ≥ 90% indicated a PaO2 ≥ 60mmHg, while highlighting that clinical factors such as pH, PaCO2 and body temperature - all well-known causes of curve shifts - could affect the accuracy of inferring these values.^(8,48)^

**Table 2:** Summarized data from the included clinical studies comparing ratio of the arterial blood oxygen saturation to the fraction of inspired oxygen and ratio of the partial pressure of arterial oxygen to the fraction of inspired oxygen

**Reference**	**Sample size (observations)**	**Population**	**Disease**	**Intervention (MV, NIV or SB)**	**Comparison between S/F and P/F**	**Outcomes**
Neonatal and pediatric clinical studies						
Khemani et al.^(6)^	1,201	Children within 7 days	NA	MV within 7 days in pediatric ICU	At D1, S/F better discriminated mortality than P/F (p = 0.0003)	S/F ≤ 150, mortality 38.3%; S/F = 150 - 221, mortality 6.0%; S/F = 221 - 265, mortality 1,5%; S/F > 265, mortality 2.6%
Thomas et al.^(7)^	255 (2,839 observations)	Children and adolescents < 21 years	ARDS	Instillation of calfactant or placebo and 102 prone *versus* supine	S/F ≤ 253 indicated P/F ≤ 300 with 93% sensitivity and 43% specificity S/F ≤ 212 indicated P/F ≤ 200 with 76% sensitivity and 83% specificity	NA
Khemani et al.^(48)^	137 (1,207 observations)	Children >27 weeks gestational age and < 18 years	Any that required MV	Controlled MV	1/S/F = 0.00232 + 0.443/P/F S/F = 221 (95%CI 215 - 226) indicating P/F = 200, with 88% sensitivity and 78% specificity in detecting P/F < 200 S/F = 264 (95%CI 259 - 269) indicating P/F = 300, with 91% sensitivity and 53% specificity in detecting P/F < 300	NA
Lobete Prieto et al.^(49)^	8 (40 observations)	Children admitted to ICU (age = 4.62 years)	Any that required intensive care	NA	S/F = 256.7 indicating P/F < 200 with 84,6% sensitivity and 85,2% specificity S/F = 297.6 indicating P/F < 300 with 89.7% sensitivity and 82% specificity	NA
Lobete et al.^(50)^	235 (1,643 observations)	Children admitted to ICU	Any that required intensive care (except cardiac surgery)	MV, NIV and SB	1/S/F = 0.00164 + 0.521/P/F (p < 0.0001, R^2^ = 0.843) S/F = 296 (95%CI 285 - 308) indicated P/F < 300, with 91% sensitivity and 87% specificity S/F = 236 (95%CI 228 - 244) indicated P/F < 200, with 88% sensitivity and 86% specificity S/F = 146 (95%CI 142-150) indicated P/F < 100, with 52% sensitivity and 99% specificity	NA
Bilan et al.^(51)^	70	Children admitted to ICU (age = 32 ± 5 months)	ARDS	MV	S/F = 235 indicated P/F < 300 with 57% sensitivity and 100% specificity S/F = 181 indicated P/F < 200 with 71% sensitivity and 82% specificity	NA
Wong et al.^(52)^	70	Pediatric ICU patients (1 day to 16 years)	ARDS	MV, NIV and SB	NA	S/F at D3: survivors: 221; nonsurvivors: 149; p = 0.006 S/F at D7: survivors: 277; nonsurvivors: 146; p = 0,002
No ARDS clinical studies						
Bass et al.^(2)^	77	Clinical stable adult patients under MV	Any that required MV	MV with PEEP ≥ 5cmH2O	Spearman r = 0.83; p < 0.0001 S/F ≤ 315 indicated P/F ≤ 3 00 with 83% sensitivity and 50% specificity and S/F ≤ 235 indicated P/F ≤ 200 with 70% sensitivity and 90% specificity = 90%	NA
Venegas Sosa et al.^(3)^	25	Adults (mean age = 37 years)	Thoracic trauma	MV	Pearson r (all with p < 0.05) At admission: r = 0.616 7 hours from admission: r = 0.68 14 hours from admission: r = 0.86 24 hours from admission: r = 0.89 31 hours from admission: r = 0.92 38 hours from admission: r = 0.90 48 hours from admission: r = 0.91	NA
Zeserson et al.^(8)^	129	Adults	Any emergency department patient	MV, NIV or SB	SpO2 ≥ 90% correlated with a PaO2 ≥ 60mmHg	NA
Namendys- Silva et al.(11)	232	ICU patients ≥16 years	Any that required ICU	MV	Used Pandharipande et al.^(10)^ for substituing P/F for S/F: S/F ≤ 512 indicating P/F ≤ 400 S/F ≤ 357 indicating P/F ≤ 300 S/F ≤ 214 indicating P/F ≤ 200 S/F ≤ 89 indicating P/F ≤ 100	Higher S/F ratio for survivors than for nonsurvivors at admission and at 48 hours of admission
Schmidt et al.^(53)^	3,767 (7,544 observations)	Adults ≥ 18 years	Any that required MV	MV	Spearman r = 0.95 and correlation coefficient = 0.72 between S/F and P/F Log10 (P/F ratio) = 1.07*Log10 (S/F ratio) - 0.15 No impact after PEEP inclusion S/F = 295 indicated P/F ≤ 300 with 99% sensitivity and 9.9% specificity	NA
Kwack et al.^(54)^	456	Adults (median age = 75 years)	NA	NA	NA	Lower S/F in patients transferred from general ward to ICU (medians 165 versus 320, p < 0.01) and in mortality versus survival groups (medians 217 versus 307, p < 0.01)
Sanz et al.^(55)^	Valencian cohort: 926 Utah cohort: 213	Adults in Valencian cohort (73 years) Utah cohort (67 years)	Pneumonia	NA	Agreement when P/F < 200: (Ellis)^(56)^ - 92%; (Rice et al.)^(4)^ - 91% Agreement when P/F < 300: (Ellis)^(56)^ - 80%; (Rice et al.)^(4)^ - 70%	NA
Tripathi et al.^(57)^	2,754 (4,439 observations)	Adults ≥18 years	General anesthesia (nonthoracic and noncardiac)	MV with PEEP	Correlation between P/F and S/F: r = 0.46, p < 0.01) significant in any PEEP Linear regression: S/F = (0.26 x P/F) + 128 S/F = 206 indicated P/F = 300 S/F = 180 indicated P/F = 200	NA
Serpa Neto et al.^(58)^	260	Adults≥18 years (mean age=63 years)	Sepsis	NA	S/F ratio = 132.27 + 0.30 × (P/F) (p < 0.0001; r = 0.487) S/F = 154 indicated P/F = 100 S/F = 241 indicated P/F = 300	HR for death according to cutoff: S/F 241 - 192: HR = 1.70 (0.77 - 3.78) S/F 192 - 154: HR = 1.64 (0.66 - 4.08) S/F < 154: HR = 2.05 (1.11 - 3.81)
Mantilla et al.^(59)^	462	Adults	Exacerbated COPD	MV, NIV and SB	NA	78.6% sensitivity and 39.2% specificity for S/F in predicting mortality
Adams et al.^(60)^	25,944 (3,505,707 observations)	Adult nonparturient (mean age 65 years)	Any that required MV	MV	S/F and P/F showed moderate (r = 0.47) correlation for measures available in same hour and strong (r = 0.68) correlation when restricted to P/F < 400 and SpO2 ≤ 96%	Proportion of time with S/F < 150 (S/F-TAR) associated with higher mortality in the first 24 hours of MV In the first 24 hours of MV: S/F-TAR 0% = 16.4% mortality S/F-TAR 91 - 100% = 70.2% mortality Each 10% increase in S/F-TAR associated with 24% increase in hospital mortality (OR = 1.24 [95%CI 1.23 - 1.26], p < 0.001)
ARDS clinical studies						
Rice et al.^(4)^	672 for derivation (2,673 observations) and 402 for validation (2,031 observations)	ARDS network trial patients: Derivation: Low VT group Validation: High PEEP versus Low PEEP groups	ARDS	MV (Low VT and high versus low PEEP)	Spearman r = 0.89; p < 0.0001 S/F = 64 + 0.84 x (P/F) Effect of PEEP on S/F ratio (p < 0.001): S/F = 129 + 0.72 x (P/F) - 4.0 x (PEEP) - 0.008 x (PEEP) x (P/F) S/F = 235 indicated P/F = 200 and S/F = 315 indicated P/F = 300	NA
Pandharipande et al.^(10)^	4728 Group 1 - 1,742 observations Group 2 - 2,986 observations, only for SpO2 ≤ 98%	Group 1 - Adults under general anesthesia for noncardiovascular or thoracic surgeries Group 2 - ARDS network trial patients: Low versus High VT	Group 1 - any surgical patient Group 2 - ARDS	MV	Spearman’s rho (p < 0,001) for SOFA with S/F and P/F: overall = 0.85 Group 1 Log (P/F)=0.48+0.78xLog(S/F) Group 2 PEEP < 8cmH2O; Log (P/F) = 0.06 + 0.94 x Log (S/F) PEEP 8 - 12cmH2O Log (P/F) =-0.13+1.01 x Log (S/F) PEEP > 12cmH2O Log (P/F) =-0.47 + 1.17 x Log (S/F)	Similar correlations between SOFA scores using P/F and S/F for ICU LOS and VFD ICU LOS *versus* SOFA respiratory using S/F: r = 0.36 (p = 0.013) VFD *versus* SOFA respiratory using S/F: r =-0.33 (p = 0.025)
Brown et al.(61)	1,184	ARDS network (EDEN, OMEGA and SAILS) trial patients	ARDS	NA	Correlation between measured and imputed P/F using S/F from: (Ellis)^(56)^, nonlinear: r = 0.84 (Rice et al.)^(4)^ linear: r = 0.733 (Pandharipande et al.)^(10)^ log-linear: r = 0.73	NA
Chen et al.(62)	101	ICU patients (mean age 69 years)	ARDS	MV	NA	Lowest S/F ratio during ICU stay (148 in survivors versus 139 in nonsurvivors) associated with mortality (p=0.046) AUC for S/F (0.616, p = 0.046) for mortality prediction AUC from P/F (0.603; p = 0.08) for mortality prediction
Chen et al.^(63)^	124	ICU patients ≥ 18 years	ARDS	NA	Used predefined cutoff of S/F < 315 for ARDS. Overall discordance between S/F and P/F for ARDS diagnosis was 8.2% (n = 30 from 362)	S/F cutoffs for ARDS severity and mortality rates: 315 - mild: 30.6% 235 - moderate: 23.1% 144 - severe: 61.1% p < 0.001
Covid-19 clinical studies						
Lu et al.^(12)^	280	Severe and critically ill COVID-19 patients	COVID-19	MV, NIV and SB	NA	Strong association between √S/F and the risk for death, corresponding to 1.82-fold increase (95%CI: 1.56-2.13) in the mortality risk
Wang et al.^(64)^	344	Severe and critically ill COVID-19 patients	COVID-19	MV, NIV and SB	NA	Negative correlation between S/F ratio and ARDS incidence (r =-0.68) - every 10 units increase in S/F correlated with 10% decrease in fatality (HR = 0.90; p < 0.001)

MV - mechanical ventilation; NIV - noninvasive ventilation; SB - spontaneous breathing; NA - not available; ICU - intensive care unit; D - day; S/F - ratio of the arterial blood oxygen saturation to the fraction of inspired oxygen; P/F - ratio of the partial pressure of arterial oxygen to the fraction of inspired oxygen; ARDS - acute respiratory distress syndrome; 95%IC: 95% confidence interval; PEEP - positive end-expiratory pressure; SpO2 - oxygen saturation; PaO2 - partial pressuare of oxygen; HR - hazard ratio; COPD - chronic obstructive pulmonary disease; TAR - time at risk; OR - odds ratio; VT - tidal volume; SOFA - Sequential Organ Failure Assessment; LOS - length of stay; VFD - ventilator-free days. Rice equation: S/F = 64 + (0.84 × (P/F)); Ellis equation: PO2 = (B + A)^1^/^3^ - (B - A)^1^/^3^, where A = 11 700(S-1 - 1)

-1 and B = (50^3^ + A^2^)^0^.^5^.

In an attempt to improve the prediction of the P/F from the S/F, researchers have used transcutaneous carbon dioxide measurements in children, with positive although preliminary results.^(49)^ In a prospective, multicenter observational study including six pediatric ICUs, a P/F value of 300 corresponded to an S/F value of 264 (95% confidence interval - 95%CI 259 - 269), while in moderate ARDS, a P/F value of 200 corresponded to an S/F value of 221 (95%CI 215 - 226).^(48)^ The relationship between S/F and P/F was better expressed by 1/S/F and 1/P/F, with a strong linear relationship using the regression equation 1/S/F = 0.00232 + 0.443/P/F.^(48)^ Furthermore, in this study, a cutoff S/F value of 221 exhibited an excellent discriminant ability for ARDS, with 88% and 78% sensitivity and specificity, respectively, for a P/F below 200.^(48)^ In critically ill children, the 1/S/F was strongly associated with the 1/P/F, yielding the equation 1/S/F = 0.000164 + 0.521/P/F.^(50)^

Another pediatric ARDS investigation suggested the following regression equation: S/F = 57 + 0.61 × P/F.^(51)^ Nonlinear equations are more accurate in predicting P/F from S/F than linear or log-linear equations.^(65)^ An epidemiological study of pediatric ARDS showed reduced ventilator-free days and ICU-free days in children with a low S/F, highlighting the association between poor S/F and worse outcomes.^(52)^

Despite its unquestionable value, notably in neonates, children, and resource-limited settings, further evidence and specific guidelines are required to support an accurate and safe use of the S/F as a surrogate for the P/F in these patients.

### Adult patients without acute respiratory distress syndrome

A Spearman’s rho of 0.66 (p < 0.001) was found when trying to predict SaO_2_ from SpO_2_ in a Spanish study of adult pneumonia patients.^(55)^ Another study in anesthetized patients obtained the regression equation S/F = (0.26 × P/F) + 128, with P/F between 300 and 200 corresponding to S/F between 206 and 180, respectively.^(57)^ Investigating ICU patients in two countries (Brazil and Netherlands), researchers described another equation for linear regression of S/F and P/F: S/F = 132.27 + 0.30 × (P/F).^(58)^ The same study showed that, in patients with septic shock, lower S/F (lowest tertile) represented increased mortality ratios (hazard ratio - HR = 2.04; 95%CIT 1.05 - 3.94%) compared to the reference group (patients in the highest tertile, with S/F above 236) and that S/F were excellent at discriminating patients with *versus* without severe hypoxemia (P/F under 100) and with *versus* without hypoxemia (P/F above 300).^(58)^ A single-center investigation of ICU patients receiving MV showed Spearman correlation coefficients of 0.83 (p < 0.05) for S/F and P/F, 83% sensitivity and 50% specificity for the S/F ≤ 315 indicating a P/F ≤ 300, and 70% sensitivity and 90% specificity for the S/F ≤ 235 indicating a P/F ≤ 200.^(2)^ In chest trauma patients, the S/F exhibits a good correlation with P/F (1 hour posttrauma: R^2^ = 0.61; 7 hours posttrauma: R^2^ = 0.68; 14 hours posttrauma: R^2^ = 0.86; 24 hours posttrauma: R^2^ = 0.89; 31 hours posttrauma: R^2^ = 0.92; 38 hours posttrauma: R^2^ = 0.90; 48 hours posttrauma: R^2^ = 0.91; p < 0.05 for all abovementioned R^2^ values).^(3)^

The S/F has also been investigated in chronic obstructive pulmonary disease (COPD). Colombian investigators reported 76.9% (95% CI 58.8 - 95%) sensitivity and 39.2% (95%CI 34.4 - 43.9%) specificity for 30-day mortality in COPD as compared to 80.8% (95%CI 63.7 - 97.8%) sensitivity and 53.2% (95%CI 48.3 - 58%) specificity when using P/F.^(59)^

One major limitation in correlating S/F and P/F, with practical consequences, is when any form of supplemental oxygen is given and the SpO2 values are above 90%. In spontaneously breathing patients, supplemental oxygen often masks the ability of pulse oximeters to detect hypoventilation, showing significantly higher desaturation in patients breathing room air (9.0 *versus* 2.3%; p = 0.02).^(66)^ Face masks and high-flow nasal cannula oxygen therapy are widely used in EDs and ORs; thus, increasing FiO2 in the steeper portion of the oxygen-Hb dissociation curve could mask ongoing gas-exchange issues. A study involving anesthetized patients showed only a moderate correlation (r = 0.46; p < 0.01) between the S/F and P/F.(57)

### Adult patients with acute respiratory distress syndrome

Although it is easier to perform ABG in adults than in neonates and children, using S/F as a surrogate for P/F could be of great value, especially in resource-limited settings. Before discussing whether S/F is a good surrogate for P/F, it is important to highlight that another index using SpO2 instead of PaO2 has been evaluated. Just as the oxygenation index has been derived for neonates,^(9)^ the so-called oxygenation saturation index (mean airway pressure × FiO2 × 100/SpO2) has been developed using SpO2 instead of PaO2 for adults. The oxygenation index and oxygenation saturation index both showed good predictive performance for ARDS mortality using Receiver Operating Characteristic (ROC) curve analysis.^(62)^

**Figure 1:**
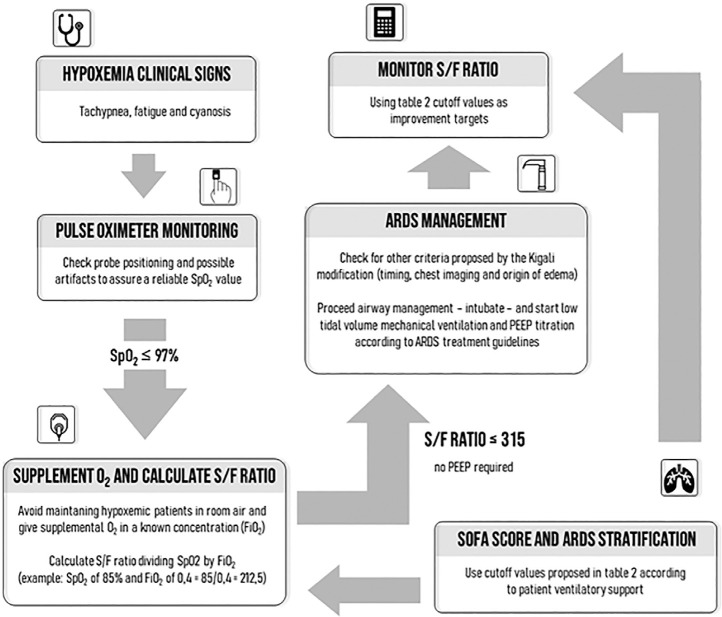
Algorithm for using the arterial blood oxygen saturation to the fraction of inspired oxygen ratio as a diagnostic and prognostic tool for acute respiratory distress syndrome in adults. SpO_2_ - arterial blood oxygen saturation; FiO2 - fraction of inspired oxygen; S/F - ratio of the arterial blood oxygen saturation to the fraction of inspired oxygen; PEEP - positive end-expiratory pressure; ARDS - acute respiratory distress syndrome.

Since ARDS definitions have been shaped in high-resource settings, applying them in facilities that lack resources is a huge challenge, given the requirement of positive pressure ventilation, ABG analysis, and chest radiographs.^(67)^ The challenge of diagnosing ARDS in resource-limited settings led researchers to investigate alternatives to ABG.^(68)^ In 2016, the Kigali modification was proposed, replacing computed tomography (CT) with lung ultrasound and the P/F with the S/F. It is remarkable that an ARDS definition applicable in resourcelimited facilities - based on simple techniques such as pulse oximetry and lung ultrasound - could definitely reduce underdiagnosis and facilitate epidemiological and clinical studies of ARDS.^(67)^ A secondary analysis of a large observational cohort study concluded that ARDS patients diagnosed by S/F had similar outcomes to patients diagnosed by P/F, indicating that the S/F could be a surrogate for ARDS diagnosis.^(63)^ In addition, a singlecenter study proposed an S/F threshold of 181 - which would correspond to a P/F of 200 - for ARDS.^(51)^ Thus, the current evidence suggests that the S/F w ARF and, notably, ARDS in resource-limited scenarios.^(50)^ Figure 1 suggests an algorithm for using the S/F as a diagnostic and prognostic tool for ARDS in adults. The clinical benefits of establishing S/F cutoff values for ARF diagnosis are clear, and S/F have been successfully tested in an automated ARDS screening tool (Spearman correlation rho = 0.72, p < 0.001).^(53)^ A recent study showed that the S/F provided superior or equal accuracy in predicting ICU transfers from the respiratory ward compared to preexisting early warning scores (Modified Early Warning Scores - MEWS, National Early Warning Scores - NEWS, and Vitalpac Early Warning Score - ViEWS).^(54)^

### Use of the ratio of the arterial blood oxygen saturation to the fraction of inspired oxygen in clinical scores

**Figure 2:**
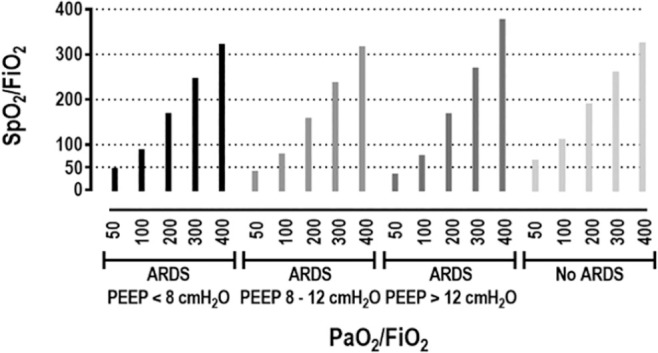
Expected arterial blood oxygen saturation to the fraction of inspired oxygen ratio according to relevant values of the ratio of the partial pressure of arterial oxygen to the fraction of inspired oxygen in different positive end-expiratory pressure levels in acute respiratory distress syndrome patients and patients without acute respiratory distress syndrome. At lower positive end-expiratory pressure levels (positive end-expiratory pressure < 8cmH_2_O), low PaO_2_/FiO_2_ (50 and 100mmHg) showed good agreement with the SpO_2_/FiO_2_ ratio, while there was an underestimation of the SpO_2_/FiO_2_ ratio at high PaO_2_/FiO_2_ (300 and 400mmHg). At higher positive end-expiratory pressure levels (positive end-expiratory pressure > 12cmH_2_O), there was an underestimation of the SpO_2_/FiO_2_ ratio at low PaO_2_/FiO_2_ (50 and 100mmHg), while at high PaO_2_/FiO_2_ (300 and 400mmHg) there was good agreement with the SpO_2_/FiO_2_ ratio. There was an underestimation of the SpO_2_/FiO_2_ ratio at high PaO_2_/FiO_2_ (300 and 400mmHg) in patients without acute respiratory distress syndrome. SpO_2_/ FiO_2_ - arterial blood oxygen saturation/fraction of inspired oxygen; PaO_2_/FiO_2_ - partial pressure of arterial oxygen/fraction of inspired oxygen; ARDS - acute respiratory distress syndrome; PEEP - positive end-expiratory pressure.

The ratio of arterial blood oxygen saturation to the P/F has been validated as a surrogate for P/F in the SOFA score. For instance, values of 89, 214, 357, and 512 corresponded to P/F of 100, 200, 300, and 400, respectively, in mechanically ventilated patients. Different positive end-expiratory pressure (PEEP) values have been shown to impact S/F. When ventilating with PEEP < 8cmH_2_O, S/F of 115, 240, 370, and 502 corresponded to P/F of 100, 200, 300, and 400, respectively. At a PEEP between 8 and 12cmH2O, the same P/F corresponded to S/F of 130, 259, 387, and 515, while at PEEP > 12cmH2O, they corresponded to S/F values of 129, 234, 332, and 425. Both the S/F and P/F correlated similarly with robust clinical endpoints, such as the ICU length of stay and the ventilator-free days, in this cohort of critically ill patients.^(10)^ Figure 2 shows the expected S/F according to the relevant values of P/F at different PEEP levels in ARDS patients and patients without ARDS, according to the log-log function between S/F and P/F provided by the study of Pandharipande et al.^(10)^, which used a relevant database from the ARDS Network.

Likewise, the S/F was successfully tested in a modified SOFA system (MEXSOFA).^(11)^ As previously described, PEEP improves ventilation/perfusion matching even though it does not interfere with the oxygen-Hb dissociation curve.

Despite the abovementioned limitations, the S/F has been shown to be a promising clinical tool. Two novel S/F-derived markers - the S/F time at risk (S/F-TAR) and respiratory rate-oxygenation (ROX) index - have been proposed. S/F-TAR displays the proportion of time within the first 24 hours of MV in which a patient has severe hypoxemia, defined by an S/F below 150. In the original study, patients with an S/F-TAR of 0% had significantly lower hospital mortality ratios than patients with a 24-hour S/F-TAR between 91% and 100% (16.4% *versus* 70.2%).^(60)^ The ROX index is defined as the S/F divided by the respiratory rate and has been investigated as a prognostic tool for intubation in patients under high flow nasal cannula (HFNC) therapy.^(69-74)^ This index is easily measured and may assist doctors in making decisions about intubation in HFNC patients since lower values indicate a higher intubation risk. Promising results have been reported in recent COVID-19 clinical studies.^(50-55)^

### COVID-19 clinical studies

Developing countries faced resource shortages long before the current pandemic. The Kigali modification for ARDS diagnosis is an excellent example of an effort to bypass these limitations. However, the COVID-19 pandemic has shown the importance of reducing medical costs to enable a massive, population-wide provision of care even in resource-rich countries. Accordingly, using S/F instead of P/F could be of great value in COVID-19 patient management. Unfortunately, there is only a small body of evidence to support this use; our literature review found only two studies on this topic. The first was a theoretical discussion of the use of smartphone-based pulse oximetry for the early detection of silent hypoxemia among COVID-19 outpatients, raising the possibility of early detection of pneumonia and consequent reductions in ICU admissions, intubations, and mortality.^(17)^ In the second study, the authors observed a sharp reduction in the S/F in nonsurvivors among COVID-19 ICU patients, highlighting a strong association between the S/F and mortality risk. The same study suggests that the S/F could represent a noninvasive prognostic marker in hospitalized COVID-19 patients.^(12)^

## FINAL COMMENTS

There is some evidence that the S/F criteria can be a surrogate for P/F in different clinical scenarios. This is reinforced by the fact that unnecessary invasive procedures should be avoided in patients with ARF, and clinical guidelines recommend continuous pulse oximetry monitoring of ARF patients.^(75)^ It is undeniable that pulse oximeters are becoming a widespread, low-cost monitoring technology; hence, replacing P/F with S/F may allow even resource-limited facilities to objectively diagnose ARF.^(1,39)^ Physicians may recognize the low value of the S/F as a single time point parameter and simultaneously use it in a longitudinal perspective and incorporate it into new indices that have shown relevance in recent clinical studies. Last but not least, the S/F may provide a zero-cost alternative for the diagnosis of ARF in COVID-19, although the limitations of pulse oximetry should always be kept in mind.
